# A guide to the identification of the terrestrial Isopoda of Maryland, U.S.A. (Crustacea)

**DOI:** 10.3897/zookeys.801.24146

**Published:** 2018-12-03

**Authors:** Jeffrey W. Shultz

**Affiliations:** 1 Department of Entomology, University of Maryland, College Park, Maryland, USA University of Maryland College Park United States of America

**Keywords:** Oniscidea, taxonomy, taxonomic key

## Abstract

The terrestrial isopod fauna of Maryland is inferred using the taxonomic literature, internet-based citizen science projects, and original collecting. Twenty-two species are either known or are likely to occur in the state. This includes 17 mostly-European adventive species that comprise the vast majority of records. Of the five expected native species, three occur in or near marine or estuarine littoral habitats and each has yet to be recorded or recorded from only a single locality. This situation likely reflects the long history of systematic work on the European fauna and the availability of keys for the identification of these taxa, which contrasts with the limited taxonomic work on native species. A taxonomic key, illustrations, and brief descriptions of species known or expected to occur in Maryland are provided.

## Introduction

There are no up-to-date, comprehensive taxonomic keys to the terrestrial isopod species of the eastern United States, the most recent being that of Muchmore (1990). A standard solution to the identification problem has been to send specimens to taxonomic experts, but such expertise is increasingly rare (Sfenthourakis and Taiti 2015). Alternatively, the internet offers several citizen-science projects: BugGuide (https://bugguide.net/), iNaturalist (https://www.inaturalist.org/), and Maryland Biodiversity Project (https://www.marylandbiodiversity.com/) where photos of specimens can be posted for identification by the internet community. Unfortunately, the photos often do not show key diagnostic features and the specimens thus remain unidentified or even misidentified. Given such problems, it has become common in ecological studies to circumvent the issue by grouping unidentified specimens into look-alike morphospecies, a practice known as “taxonomic minimalism” (Beattle and Oliver 1994), which limits comparisons across studies and may inadvertently inflate or reduce the actual number of species in a study (Krell 2004). This “solution” hampers the ability to associate taxonomic species with particular localities or habitats, to understand the dynamics of communities, and to recognize new native, adventive, or invasive species. The goal of this contribution is to provide a taxonomic key to the 22 species of terrestrial isopods that are known or expected to occur in Maryland as a step toward reducing the need for taxonomic minimalism in ecological studies in the state and adjacent areas.

Most terrestrial isopod species in Maryland are widely distributed and common European adventives (e.g., Hornung and Szlávecz 2003, Hornung et al. 2015, Norden 2008). Consequently, identification tools designed for use in Europe (e.g., Hopkin 2014) may be useful in the eastern United States and can potentially identify native forms to genus (e.g., *Ligidium*, *Miktoniscus*). Still, because such keys deal with a more diverse fauna than exists in Maryland and may omit some European species, such as *Chaetophilosciasicula* (Hornung and Szlávecz 2003), the potential for misidentification is increased.

Maryland’s native terrestrial isopod fauna is small and probably consists of five species. Three marine or estuarine littoral species are either known from the state, *Mitkoniscusspinosus* (Say, 1818) (Trichoniscidae) and *Scyphacellaarenicola* Smith, 1873 (Scyphacidae), or are expected to occur based on established distributions, *Littorophilosciavittata* (Say, 1818) (Halophilosciidae). Two inland terrestrial species are also expected, *Miktoniscusmedcofi* (Van Name, 1940) (Trichoniscidae) and *Ligidiumelrodii* (Packard, 1873) (Ligiidae), with the first state record of *L.elrodii* reported here. In addition, *M.spinosus* has been found in moist inland habitats in North Carolina (Schultz 1976) and this could be the case in Maryland. In fact, a recent photo taken at a suburban park in the District of Columbia, which is essentially encompassed geographically by Maryland, appears to show a specimen of *Miktoniscus* sp. (BugGuide: https://bugguide.net/node/view/1469429). The paucity of state records for native terrestrial isopods likely reflects several factors, including the lack of taxonomic work on the group, substantial reduction in native habitat, and the limited accessibility of taxonomic keys and other methods of identification.

## Basic external anatomy of terrestrial isopods

**Figure 1.**
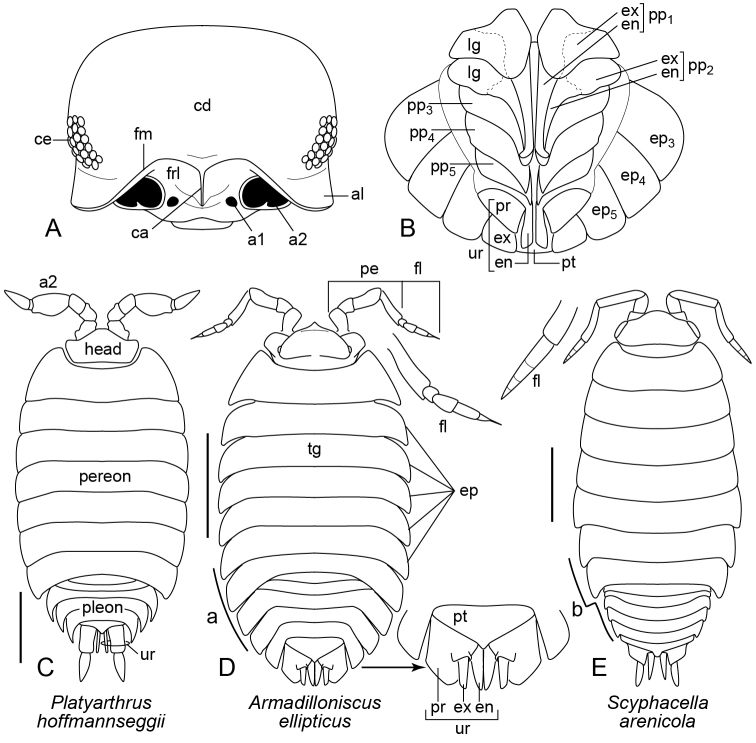
**A***Cylisticusconvexus*, head, dorsoanterior view, antennae removed (based on Schmidt 2008: figure 32) **B***Armadillidiumvulgare*, male pleon, ventral view **C***Platyarthrushoffmannseggii* (after Hopkin 2014: figure 17) **D***Armadilloniscusellipticus*, insets highlight uropods and antennal flagellum **E***Scyphacellaarenicola*, inset highlights antennal flagellum. Abbreviations: **a** lateral perimeter narrows gradually from pereon to pleon; **a1** socket of antenna I; **a2** antenna II or socket of antenna II; **al** anterolateral lobe; **b** lateral perimeter narrows abruptly from pereon to pleon; **ca** carina; **cd** cephalic dorsum; **ce** compound eye; **en** endopodite; **ex** exopodite; **fl** flagellum; **fm** frontal margin; **frl** frontal lamina; **lg** lung; **tg** tergite; **pe** peduncle of antenna II; **pp** pleopod; **pr** protopodite; **pt** pleotelson; **ur** uropod. Scale bars: 1 mm.

The isopod body has three main regions or tagmata: head (cephalothorax), pereon (thorax), and pleon (abdomen) (Figure 1C). The anterior part of the head is typically divided transversely by a raised frontal margin (fm, Figure 1A) that begins on the lateral surface, passes anteriorly ventral to the eye region and dorsal to the base of the antennae, and traverses the anterior surface of the head. It separates the cephalic dorsum (or vertex) (cd, Figure 1A) from the frontal lamina (frl, Figure 1A), a sclerotized region bordered laterally by the bases of the two large antennae. In some cases the frontal margin is interrupted medially or is absent, with the latter obscuring the distinction between the cephalic dorsum and frontal lamina. The frontal margin is often produced into a pair of anterolateral lobes (al, Figure 1A) between the eye region and base of the antenna. The frontal margin may also be produced medially in various ways (e.g., Figs 1A, 4A, C–F).

**Figure 2.**
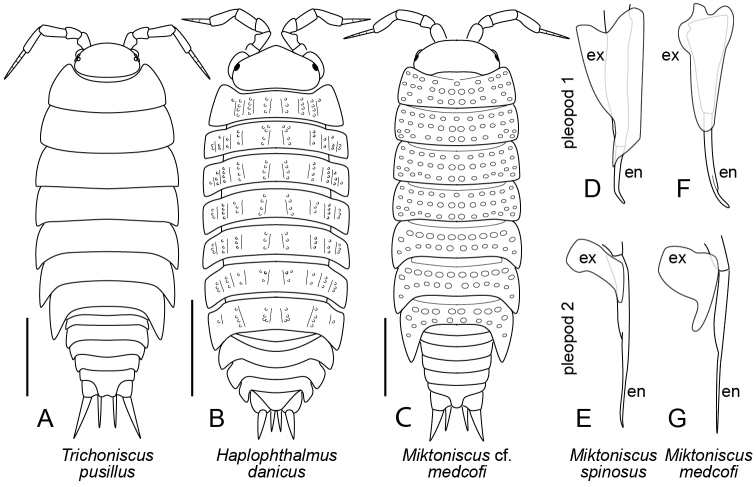
Trichoniscidae. **A***Trichoniscuspusillus***B***Haplophthalmusdanicus*, highlighting cuticular sculpture of pereon **C**Miktoniscuscf.medcofi, highlighting cuticular sculpture of pereon **D–E***Miktoniscusspinosus*, male **D** right pleopod I, ventral view **E** right pleopod II, ventral view (based on Schultz 1976: figs 8, 9, as *M.halophilus*) **F–G***Miktoniscusmedcofi*, male: **F** right pleopod I, ventral view **G** right pleopod II, ventral view (based on Schultz 1976: figs 40, 41). Abbreviations: **en** endopodite; **ex** exopodite. Scale bars: 1 mm.

**Figure 3.**
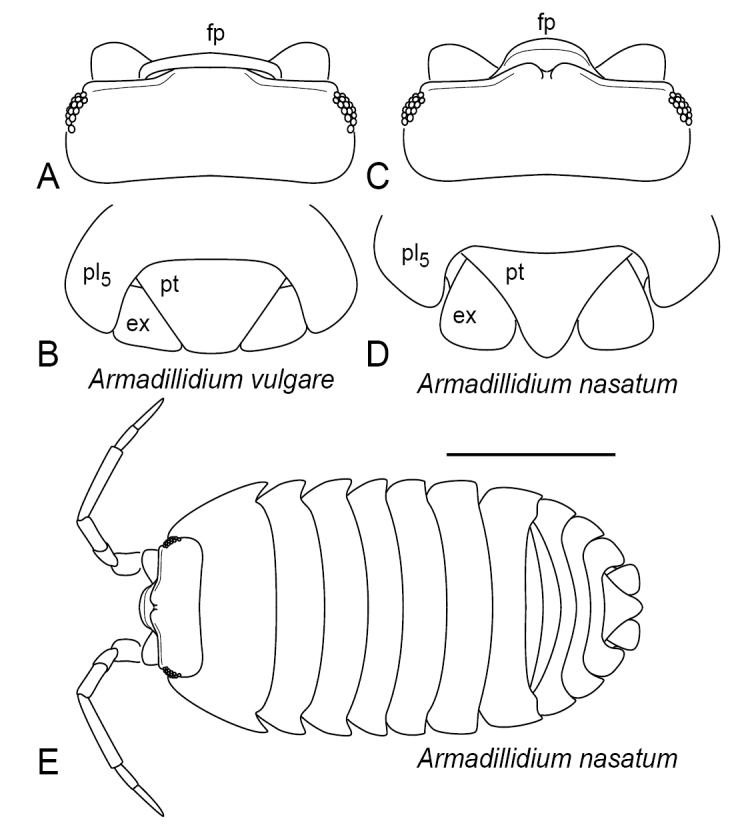
Armadillidiidae. **A–B***Armadillidiumvulgare***A** head, dorsal view **B** posterior end of pleon, dorsal view **C–E***Armadillidiumnasatum***C** head, dorsal view **D** posterior end of pleon, dorsal view **E** Dorsal view. Abbreviations: **ex** exopodite of uropod; **fp** frontal projection; **pl_5_** pleonal tergite V; **pt** pleotelson. Scale bar: 5 mm.

**Figure 4.**
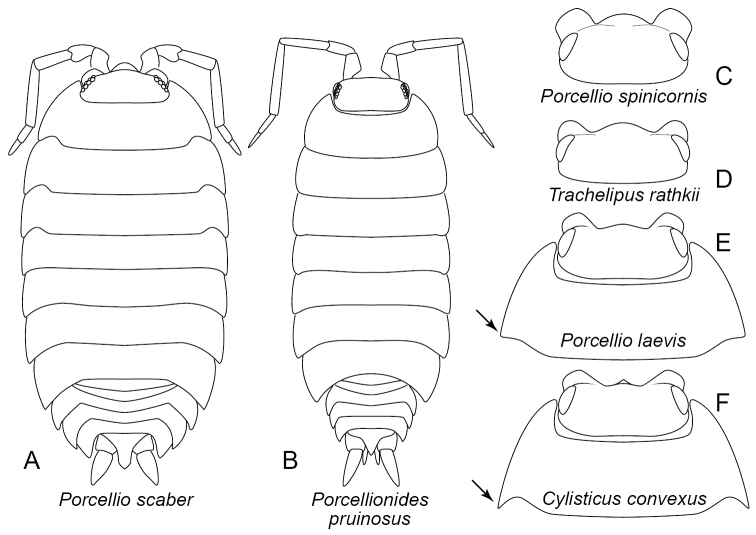
Porcellionidae, Trachelipodidae, Cylisticidae. **A***Porcellioscaber***B***Porcellionidespruinosus***C***Porcelliospinicornis*, head, dorsal view **D***Trachelipusrathkii*, head, dorsal view **E***Porcelliolaevis*, head and first pereonal tergite, dorsal view **F***Cylisticusconvexus*, head and first pereonal tergite, dorsal view.

**Figure 5.**
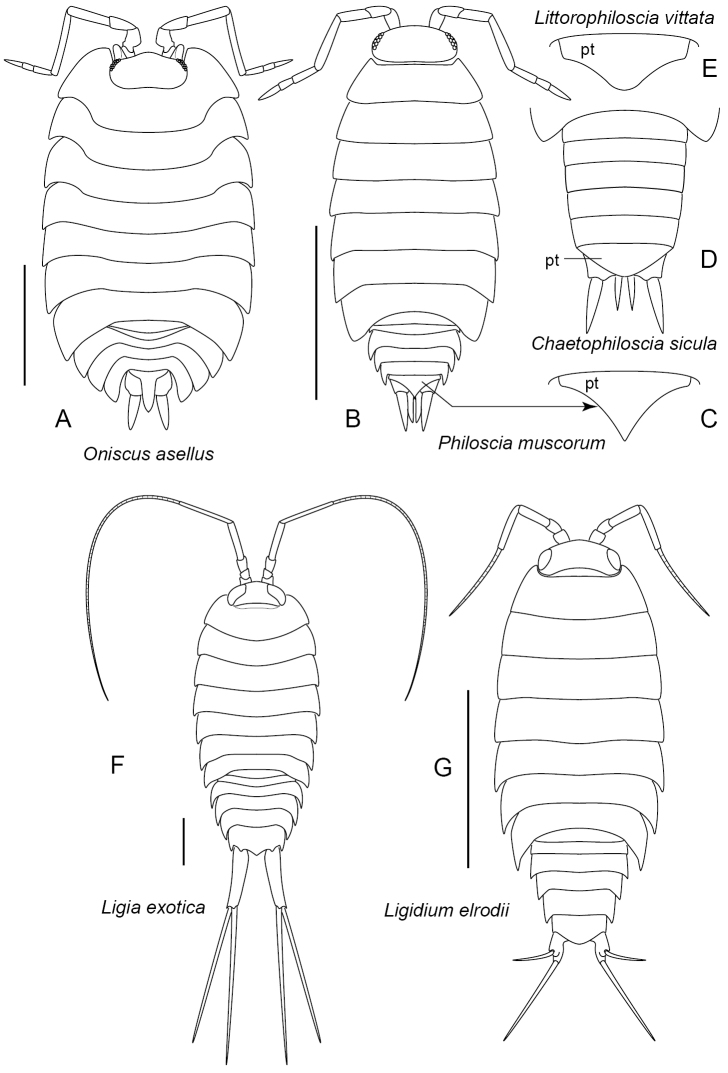
Oniscidae, Philosciidae, Halophilosciidae, Ligiidae. **A**Oniscidae: *Oniscusasellus***B–E**Philosciidae**B–C***Philosciamuscorum***B** dorsal view **C** pleotelson **D***Chaetophilosciasicula*: dorsal view of pleon (after Vandel 1962: figure 247) **E***Littorophilosciavittata*: pleotelson, dorsal view **F–G**Ligiidae**F***Ligiaexotica***G***Ligidiumelrodii*. Scale bars: 5 mm.

Each eye consists of either a compound eye (ce, Figure 1A) or one to three ocelli and is absent in some species (e.g., Figure 1C). Antennules (= antennae I) are very small and positioned medial to the base of the large antennae (antenna II). The five proximal articles (antennomeres) of the antennae constitute the peduncle (pe, Figure 1D), with the distal-most article bearing a terminal flagellum (fl, Figure 1D). Most species have only two or three distinct flagellar articles (Figs 3E, 4A, B, 5A, B) but some have 10 or more (Figure 5F, G). Species in the family Trichoniscidae have what can appear to be a thin, tapering undivided flagellum (Figure 2A–C), but inspection with high magnification will reveal multiple articles, the number of which can be useful for species identification.

The pereon consists of seven broad segments (Figure 1C), each bearing a pair of legs (pereopods) ventrally. The dorsal surface of each segment, the tergite (tg, Figure 1D), may be smooth or armed with tubercles or other sculpture (e.g., Figure 2B, C). The tergites bear ventrolateral extensions (epimera) (ep, Figure 1D) that collectively form the protected ventral space within which the legs operate.

The pleon has five free segments. The lateral margins of the first two lack epimera. The remaining three may be abruptly narrower than the last pereonal tergite (b, Figure 1E), often due to the absence or reduction of epimera. However, when large epimera are present, the pleonal margin may appear as a smooth continuation of the pereonal margin (a, Figure 1D).

Each free pleonal segment has a pair of ventral appendages, the pleopods, with a large plate-like part, the exopodite, and a medial part, the endopodite (Figure 1B). The endopodites of the first two pairs are enlarged and elongated in males (en, Figure 1B) and bear species-specific modifications (e.g., Figure 2D–G). The exopodites may have air-filled respiratory tubules called pseudotracheae that function as a lung. The lungs are visible in live specimens as thickened white patches on the lateral part of the exopodites. The white color typically disappears through loss of air when the animal is preserved. In species found in Maryland, the lungs may be absent, present in the first two pairs of pleopods (lg, Figure 1B) or present in all pleopods.

The last apparent segment of the pleon is the pleotelson, a combination of a terminal segment and the telson (pt, Figs 1B, D, 3B, D, 5C–E). The pleotelson has a pair of uropods, each comprising a basal protopodite that articulates distally with a medial endopodite and lateral exopodite (Figure 1B–D). The relative sizes, lengths, and shapes of these elements and their position with respect to the pleotelson are often useful in identification.

### Key to adult terrestrial isopods of Maryland

**Table:** 

1	Cuticle translucent to white. Eyes absent. Antenna with antennomere V much wider than the other antennomeres (Figure 1C). Associated with ants. (Platyarthridae)	***Platyarthrushoffmannseggii* Brandt, 1833**
–	Cuticle color variable. Eyes present, each usually compound or with one or three ocelli. Antenna variable, but antennomere V not significantly wider than the others. Not associated with ants or associated with ants only incidentally	**2**
2	Antennal flagellum comprising two to many distinct articles (Figs 1D, 3E, 4A, B, 5A, B, F, G)	**3**
–	Antennal flagellum superficially appearing to be one article, either robust (fl, Figure 1E) or thin, tapering and elongate (Figure 2A–C), but comprising up to six articles visible with high magnification	**4**
3	Antenna with two distinct flagellar articles (Figs 3E, 4A, B). Uropods may extend beyond elliptical perimeter of the body in dorsal view (Figure 4A, B) or may not (Figure 3B, D)	**9**
–	Antenna with three distinct flagellar articles (Figure 5A, B). Uropods extending beyond elliptical perimeter of body in dorsal view; protopodite usually not extending beyond pleotelson; exopodite large, conical or spear-head shaped; endopodite thin (Figure 5A, B, D)	**16**
–	Antenna with four distinct flagellar articles (Figure 1D). Uropods completing posterior elliptical perimeter of body in dorsal view, protopodite large, dorsoventrally flattened; exopodite small, endopodite elongate (Figure 1D). (Detonidae)	***Armadilloniscusellipticus* (Harger, 1878)**
–	Antenna with flagellum long, comprising 10 or more flagellar articles (Figure 5F, G). Uropods extending beyond elliptical perimeter of body in dorsal view; with protopodite robust, projecting posteriorly beyond pleotelson; endopodite and exopodite thin, elongate (Figure 5F, G). (Ligiidae)	**19**
4	Compound eye present. Antennal flagellum approx. the same width basally as antennomere V and comprising four articles that decrease in size distally (fl, Figure 1E). Sandy marine beaches. (Scyphacidae)	***Scyphacellaarenicola* Smith, 1873**
–	Compound eye absent, but with one or three ocelli. Antennal flagellum abruptly narrower than preceding article, a narrow tapering cone (Figure 2A–C) comprising up to six articles visible with high magnification. Not usually associated with sandy marine beaches. (Trichoniscidae)	**5**
5	Surface of pereon smooth; without tubercles, denticles or other sculpture (Figure 2A)	**6**
–	Surface of pereon sculptured, with tubercles and/or denticles (Figure 2B, C)	**7**
6	Each eye with three ocelli. Antenna with four or five flagellar articles visible with high magnification. Body length up to 5 mm (Figure 2A)	***Trichoniscuspusillus* Brandt, 1833**
–	Each eye with one ocellus. Antenna with six flagellar articles visible with high magnification. Body similar to Figure 2A, length up to 7 mm	***Hyloniscusriparius* (C. L. Koch, 1838)**
7	Pereon with tubercles or denticles on longitudinal ridges (Figure 2B). Pleon not abruptly narrower than pereon; pleonal tergites with prominent epimera. Cuticle translucent or white, without dark pigments. Antenna with three flagellar articles visible with high magnification	***Haplophthalmusdanicus* Budde-Lund, 1880**
–	Pereon with tubercles in transverse or roughly transverse rows (Figure 2C). Pleon abruptly narrower than pereon. Cuticle pigmented. Antenna with four flagellar articles visible with high magnification. (*Miktoniscus*)	**8**
8	Male pleopod I (Figure 2D) with exopodite long, almost as long as endopodite, ending in point; tip of endopodite long, round in cross section. Near marine or estuarine grasses, debris, etc. but may also occur in moist inland habitats	***Miktoniscusspinosus* (Say, 1818)**
–	Male pleopod I (Figure 2F) with exopodite shorter, approx. half the length of endopodite, tapering distally but terminus broadly rounded; endopodite long, flattened in cross section. Moist terrestrial habitats	***Miktoniscusmedcofi* (Van Name, 1940)**
9	Exopodite of uropod not extending beyond elliptical perimeter of body in dorsal view; broad, dorsoventrally flattened; protopodite and endopodite largely hidden in dorsal view (Figure 3B, D, E, cf. 1B). In life, able to roll into a ball with antennae hidden. (Armadillidiidae: *Armadillidium*)	**10**
–	Exopodite of uropod extending beyond elliptical perimeter of body in dorsal view; protopodite visible in dorsal view; exopodite prominent, attaching at terminus of protopodite, broad basally, tapering distally; endopodite thin, attaching at medial base of protopodite (Figure 4A, B). In life, unable to roll into a ball or, if able to enroll, antennae remain exposed	**11**
10	Head with median frontal projection extending dorsally, with dorsal margin overlapping anterior edge of cephalic dorsum (fp, Figure 3A). Pleotelson subtrapezoidal, with posterior margin nearly straight (pt, Figure 3B)	***Armadillidiumvulgare* (Latreille, 1804)**
–	Head with prominent, median frontal projection extending dorsoanteriorly and not overlapping anterior edge of cephalic dorsum (fp, Figure 3C). Pleotelson subtriangular with posterior apex variably produced, but typically with rounded terminus (pt, Figure 3D)	***Armadillidiumnasatum* Budde-Lund, 1885**
11	Pleon abruptly narrower than pereon (Figure 4B). Anterolateral lobes very small. Cuticle often with frosted-gray or dusty appearance, but this may be absent. Antennomeres IV and V usually with white terminal band. Frontal margin without evident median projection. (Porcellionidae, in part)	***Porcellionidespruinosus* (Brandt, 1833)**
–	Pleon not abruptly narrower than pereon (Figure 4A). Anterolateral lobes prominent (Figure 4A, C–F). Cuticle without frosted-gray appearance. Antenna color variable but antennomeres IV and V usually without terminal white bands. Frontal margin with some form of median projection (Figure 4A, C–F)	**12**
12	Dorsal surface of pereon essentially smooth	**13**
–	Dorsal surface of pereon with numerous bumps or tubercles	**14**
13	Posterolateral margin of first pereonal tergite produced posteriorly into pointed angle (arrow, Figure 4F). Five pairs of lungs present. Frontal margin of head with small median triangular projection (Figure 4F) corresponding to vertical median carina of frontal lamina (ca, Figure 1A). In life, capable of rolling into a ball with antennae exposed. (Cylisticidae)	***Cylisticusconvexus* (De Geer, 1778)**
–	Posterolateral angle of first pereonal tergite bluntly rounded, not produced posteriorly into a pointed angle (arrow, Figure 4E). Lungs restricted to pleopods I and II. Frontal margin with broad, convex median projection (Figure 4E). In life, not capable of rolling into a ball. (Porcellionidae, in part)	***Porcelliolaevis* Latreille, 1804**
14	Anterolateral lobes very prominent and broad, width of each approx. one-third width of head; in dorsal view, junction between median projection and anterolateral lobes V-shaped or nearly so (Figure 4C). Head dark brown to black, contrasting with base color of pereon. Pereon with dark mid-dorsal stripe (sometimes broken), usually bordered laterally by bright yellow markings that may be lost in preservative. (Porcellionidae, in part)	***Porcelliospinicornis* Say, 1818**
–	Anterolateral lobes not so wide, joining convex median projection via curved margin, not V-shaped notches (Figure 4A, D). Head color not usually contrasting with base color of pereon. Pereon with dorsal color variable; if dark median line present, then not bordered by bright yellow markings	**15**
15	Five pairs of lungs. Dorsum of pereon with low, irregular bumps and tubercles; surface usually with pattern of dark brown, reddish brown and tan; lateral surface at base of epimera with tan to nearly-white patches creating a pair of broken lines. Frontal margin with broad convex median projection (Figure 4D). (Trachelipodidae)	***Trachelipusrathkii* (Brandt, 1833)**
–	Lungs restricted to pleopods I and II (as in Figure 1B). Dorsum of pereon tuberculate; color variable, ranging from solid brown or gray to various patterns, sometimes similar to *Trachelipus* but usually without a pair of broken light lines at base of epimera. Frontal margin with prominent triangular to subtrangular median projection with rounded apex (Figure 4A). (Porcellionidae, in part)	***Porcellioscaber* Latreille, 1804**
16	Head with prominent anterolateral lobes. Pleon not abruptly narrower than pereon, body broad and distinctly elliptical in dorsal view (Figure 5A). (Oniscidae)	***Oniscusasellus* Linnaeus, 1758**
–	Head without anterolateral lobes. Pleon abruptly narrower than pereon, body more elongate, oblong in dorsal view (Figure 5B).	**17**
17	Found in vegetation or under objects near marine or brackish water. Pleotelson with lateral margins weakly concave, posterior apex bluntly rounded to truncate (pt, Figure 5E). Head with color similar to that of pereon; pereon often with dark mid-dorsal line bordered laterally by bright yellow splotches. (Halophilosciidae)	***Littorophilosciavittata* (Say, 1818)**
–	Found in terrestrial environments. Pleotelson with lateral margins essentially straight (pt, Figure 5D) or distinctly concave (pt, Figure 5C). Coloration differing from above. (Philosciidae)	**18**
18	Pleotelson triangular, with lateral margins straight or nearly so in dorsal view, posterior apex blunt (pt, Figure 5D). Pleon rather elongate, sides straight in dorsal view, epimera not forming lateral serration. Dorsal coloration of pereon purple-brown with small, light longitudinal markings (lineoles), typically without distinct mid-dorsal stripe; head color similar to pereon. Thus far known in Maryland only from forests in Baltimore	***Chaetophilosciasicula* Verhoeff, 1908**
–	Pleotelson with lateral margins distinctly concave, posterior apex pointed (pt, Figure 5B,C). Pleon more compact, epimera giving sides a serrate appearance in dorsal view. Dorsal coloration of pereon highly variable, but often brown with pattern of lighter markings, usually with very dark mid-dorsal line; head dark brown to black, often contrasting with lighter pereon. Widespread. (Philosciidae)	***Philosciamuscorum* (Scopoli, 1763)**
19	Uropod with protopodite very long, surpassing posterior terminus of pleotelson by more than the length of pleotelson; endopodite and exopodite long and thin, about equal in length, both arising from tip of protopodite (Figure 5F). Marine or brackish shorelines, splash zone, and directly adjacent areas. Larger, up to 4.8 cm	***Ligiaexotica* Roux, 1828**
–	Uropod with protopodite exceeding posterior tip of pleotelson by about one length of the pleotelson or less; endopodite about 1.5 times the length of exopodite, endopodite arising from protopodite proximal to exopodite (Figure 5G). Wet litter in woodlands, wetlands, near streams, etc. in mountains. Smaller, up to 1 cm	***Ligidiumelrodii* (Packard, 1873)**

## Family and species summaries

### Family Armadillidiidae (*Armadillidium*) (Figs 1B, 3A–E)

Length up to 15 mm. Compound eyes present. Frontal lamina with broad projection [scutellum] (fp, Figure 3A, C). Frontal margin interrupted medially (Figure 3C) but this is hidden by scutellum in *A.vulgare* (Figure 3A). Anterolateral lobes prominent. Antenna with two distinct flagellar articles (Figure 3E). Pleon not abruptly narrower than pereon (Figure 3E). Lungs limited to first two pairs of pleopods (lg, Figure 1B). Uropod with exopodite broad, dorsoventrally flattened, completing rounded posterior outline of body in dorsal view (ex, Figure 3B, D, E); protopodite and endopodite, largely hidden in dorsal view (Figs 1B, 3B, D). In life, capable of rolling into a ball with antennae hidden.

*Armadillidiumvulgare* (Latreille, 1804) (Figs 1B, 3A, B)

With features of the family and genus, also the following: Projection of frontal lamina triangular, with broad, transverse dorsal margin overlapping frontal margin (fp, Figure 3A). Pleotelson subtrapezoidal, broadly truncated posteriorly (pt, Figure 3B). Sources: Richardson (1905), Schultz (1982) and Hopkin (2014). U.S. Distribution: Introduced. Throughout contiguous 48 states (Jass and Klausmeier 2000, 2001). Recorded in Maryland by Richardson (1905), Hornung and Szlávecz (2003), Norden (2008), BugGuide, iNaturalist, and Maryland Biodiversity Project. Habitat: Synanthropic. Litter and under stones or other objects, occurs in somewhat drier conditions than most terrestrial isopods.

*Armadillidiumnasatum* Budde-Lund, 1885 (Figure 3C)

With features of the family and genus, also the following: Projection of frontal lamina extending dorsoanteriorly, not overlapping anterior margin of cephalic dorsum (Figure 3C). Pleotelson subtriangular, with rounded to somewhat pointed posterior apex (pt, Figure 3D). Sources: Richardson (1905), Schultz (1982), and Hopkin (2014). U.S. Distribution: Introduced. Eastern and central states, also Idaho and California (Jass and Klausmeier 2000, 2001). Recorded in Maryland by Smith and Goodhue (1945), Hornung and Szlávecz (2003), Norden (2008), Hornung et al. (2015), BugGuide, iNaturalist, and Maryland Biodiversity Project. Habitat: Synanthropic. Litter and under stones or other objects, often in drier habitats than most terrestrial isopods.

### Family Cylisticidae (*Cylisticus*)

*Cylisticusconvexus* (De Geer, 1778) (Figs 1A, 4F)

Body length up to 15 mm. Compound eyes present. Antenna with two distinct flagellar articles. Frontal lamina divided by median vertical ridge (carina) that terminates dorsally as a small, triangular median projection at frontal margin (ca, Figure 1A). Anterolateral lobes prominent (al, Figs 1A, 4F). Posterolateral margin of first pereonal tergite produced into broad, posteriorly pointed angle (arrow, Figure 4F), thus distinguishing it from *Porcelliolaevis* (arrow, Figure 4E). Surface of pereon smooth. Pleon not abruptly narrower than pereon (as in Figure 4A). Five pairs of lungs. In life, capable of rolling into a ball, with antennae exposed. Sources: Richardson (1905), Schultz (1982) and Hopkin (2014). U.S. Distribution: Introduced. Throughout most of contiguous 48 states (Jass and Klausmeier 2000, 2001). Recorded in Maryland by Hornung and Szlávecz (2003), Norden (2008), Hornung et al. (2015), BugGuide, and Maryland Biodiversity Project. Habitat: A variety of moist litter and soil habitats.

### Family Detonidae (*Armadilloniscus*)

*Armadilloniscusellipticus* (Harger, 1878) (Figure 1D)

Body length up to 4 mm. Compound eye present, each with five to ten facets. Antenna with four distinct flagellar articles (fl, Figure 1D), an additional minute terminal article may be present. Pleon not abruptly narrower than pereon (a, Figure 1D). Lungs absent. Uropods with protopodite greatly enlarged, flattened, projecting posteriad far beyond pleotelson, resembling adjacent tergal extension; exopodite short, arising from dorsomedial margin of protopodite; endopodite long, arising from base of protopodite; protopodite, exopodite (ex) and endopodite (en) all ending posterior to pleotelson (pt) (Figure 1D). Sources: Harger (1880), Richardson (1905) and Schultz (1982). U.S. Distribution: Probably introduced. Massachusetts, New York, North Carolina, Florida (Jass and Klausmeier 2000, 2001), and Alabama (BugGuide). Not yet recorded from Maryland. Scattered, worldwide distribution (Schmalfuss 2003). Habitat: Drift line on marine shores, especially under planks, stones, vegetation, etc. Clings to undersurface of objects, usually does not run when disturbed (Schultz 1982).

### Family Halophilosciidae (*Littorophiloscia*)

*Littorophilosciavittata* (Say, 1818) (Figure 5E)

Body length up to 5 mm. Compound eyes present. Head without anterolateral lobes. Antenna with three distinct flagellar articles (as in Figure 5B). Pleon abruptly narrower than pereon (as in Figure 5B). Lungs absent. Unable to roll into a ball. Head color usually similar to that of pereon. Pereon and pleon usually with dark median stripe bordered by bright yellow patches, also a series of lateral submarginal yellow markings. Pleotelson with lateral margins weakly concave, posterior end rounded to almost truncate (pt, Figure 5E). Sources: Schultz (1963, 1974). U.S. Distribution: Native. Coasts of Atlantic Ocean and Gulf of Mexico, New York to Texas (Jass and Klausmeier 2000, 2001), not yet recorded from Maryland. Habitat: Marine and brackish shores between water and drift line, but also somewhat farther away from shore under objects, in marsh grass, debris, etc. (Schultz 1974).

### Family Ligiidae (*Ligia*, *Ligidium*) (Figure 5F, G)

Compound eyes present, very large with many facets. Antenna with many (> 10) flagellar articles. Uropod with protopodite projecting posteriorly beyond tip of pleotelson; endopodite and exopodite long and thin. Lungs absent. Rapid runners.

*Ligiaexotica* Roux, 1828 (Figure 5F)

Only marine littoral species known in Maryland with features of the family, also the following: Body length (excluding uropods) up to 30 mm. Antenna, especially flagellum, very long with more than 20 flagellar articles. Pleon not abruptly narrower than pereon, outline of body fusiform, tapering posteriorly. Uropod with protopodite very long, cylindrical; endopodite and exopodite nearly equal in length, attaching to protopodite at its terminus, total length of uropod up to 18 mm. Sources: Richardson (1905) and Schultz (1982). U.S. Distribution: Introduced. Known from New Jersey, North and South Carolina, Texas, Florida and California (Jass and Klausmeier 2000, 2001). Recorded from Maryland (Chesapeake Bay) by BugGuide, iNaturalist, and Maryland Biodiversity Project. Habitat: Marine to brackish shoreline, especially adjacent surfaces (pilings, rocks, etc.)

*Ligidiumelrodii* (Packard, 1873) (Figure 5G)

Only terrestrial species to occur in Maryland with features of the family, also the following: Body length up to 10 mm. Antenna with approx. 11 flagellar articles. Uropod with protopodite extending distad to pleotelson; endopodite articulating with protopodite at a point distad to articulation with exopodite, endopodite almost 1.5 times longer than exopodite. Sources: Richardson (1905) and Schultz (1970, 1982). U.S. Distribution: Native. Known from localities throughout the eastern states, also California [?] (Jass and Klausmeier 2000, 2001). Recently recorded from forests in Garrett County (J. Shultz, original observation). Habitat: Moist to wet litter and moss especially near swamps, marshes, springs, ponds, streams, also caves.

### Family Oniscidae (*Oniscus*)

*Oniscusasellus* Linnaeus, 1758 (Figure 5A)

Body length up to 16 mm. Perimeter a wide ellipse in dorsal view. Compound eyes present. Antenna with three distinct flagellar articles. Head with prominent anterolateral lobes. Pleon not abruptly narrower than pereon. Lungs absent. Pleotelson elongate, pointed. Dorsoventrally flattened, not capable of rolling into a ball. Sources: Richardson (1905), Schultz (1982), and Hopkin (2014). U.S. Distribution: Introduced. Scattered eastern and central states, also Washington and Oregon (Jass and Klausmeier 2000, 2001). Recorded from Maryland by Norden (2008), BugGuide, iNaturalist, and Maryland Biodiversity Project. Habitat: Leaf litter, under bark and similar situations.

### Family Philosciidae (*Chaetophiloscia*, *Philoscia*) (Figure 5B–D)

Compound eyes present. Antenna with three distinct flagellar articles (Figure 5B). Head without anterolateral lobes. Pleon abruptly narrower than pereon (Figure 5B, D). Lungs absent. Unable to roll into a ball.

*Chaetophilosciasicula* Verhoeff, 1908 (Figure 5D)

With features of the family, also the following: Body length up to 7.5 mm. Body outline similar to Figure 5B, but pleon more elongate and lateral margins smooth, not serrated (Figure 5D). Pleotelson broad, triangular (width about double the length) with lateral margins straight or nearly so, posterior apex bluntly rounded (pt, Figure 5D). Dorsum with purplish-brown pigment interrupted by median series of small light markings and over much of surface by light longitudinal markings (lineoles); epimera pigmented except for distal light marginal line and proximal light mark that widens anteriorly; head with markings similar to pereon. Source: Vandel (1962). U.S. Distribution: Introduced. Known thus far only from Baltimore, Maryland (Hornung and Szlávecz 2003). Habitat: In Baltimore, litter in mature temperate deciduous forest.

*Philosciamuscorum* (Scopoli, 1763) (Figure 5B, C)

With features of the family, also the following: Body length up to 12 mm. Pleon compact with epimera forming serrated lateral margin (Figure 5B). Pleotelson with lateral margins concave, posterior apex pointed (pt, Figure 5C). Head typically dark, often contrasting with lighter pereon. Pereon usually with dark mid-dorsal stripe. Sources: Schultz (1974) and Hopkin (2014). U.S. Distribution: Introduced. New England, New York, New Jersey; also Washington State (Jass and Klausmeier 2000, 2001). Recorded from Maryland by Schultz (1974), Hornung and Szlávecz (2003), Norden (2008), Hornung et al. (2015), BugGuide, iNaturalist, and Maryland Biodiversity Project. Habitat: A variety of mesic terrestrial habitats.

### Family Platyarthridae (*Platyarthrus*)

*Platyarthrushoffmannseggii* Brandt, 1833 (Figure 1C)

Body length up to 5 mm. Cuticle white to translucent. Eyes absent. Antenna with pre-flagellar antennomere very robust, wider than other peduncular antennomeres; flagellum superficially appearing to be undivided but actually with two articles, basal article very short. Anterolateral lobes prominent. Pleon not abruptly narrower than pereon. Lungs absent. Source: Hopkin (2014). U.S. Distribution: Introduced. Recorded from New York, Connecticut (Jass and Klausmeier 2000, 2001) and Philadelphia, Pennsylvania (BugGuide). Not yet reported from Maryland, but the record from Philadelphia indicates that it may occur in the state. Habitat: Soil, associated with ants.

### Family Porcellionidae (*Porcellio*, *Porcellionides*) (Figure 4A–C, E)

Compound eyes present. Antennae with two distinct flagellar articles (Figure 4A, B). Lungs restricted to pleopods I and II. Uropods extending posteriad beyond end of pleotelson (Figure 4A, B).

*Porcelliolaevis* Latreille, 1804 (Figure 4E)

With features of the family, also the following: Body length up to 18 mm. Large, broad species, body outline similar to Figure 4A. Medial frontal margin projecting anteriorly as a broad weakly convex edge. Anterolateral lobes prominent, each less than third the width of head. Pereon smooth, without tubercles or other significant sculpture. Pleon not abruptly narrower than pereon (as in Figure 4A). Posterolateral angle of first tergite bluntly rounded, not produced posteriorly (arrow, Figure 4E). Source: Hopkin (2014). U.S. Distribution: Introduced. Widely distributed in the contiguous 48 states (Jass and Klausmeier 2000, 2001). First records from Maryland are vacant lots in Baltimore City (K. Szlávecz, unpublished observation) as well as agricultural fields in Anne Arundel and Prince Georges Counties and forests in Garrett County (J. Shultz, original observation). Habitat: Diverse environments, especially synanthropic habitats.

*Porcellioscaber* Latreille, 1804 (Figure 4A)

With features of the family, also the following: Body length up to 17 mm. Head with frontal margin produced medially to form a prominent triangular to subtriangular projection. Dorsal surface of head and pereon with numerous tubercles. Pleon not abruptly narrower than pereon. U.S. Distribution: Introduced. Widely distributed in the northeastern, north-central, and western states, also Florida (Jass and Klausmeier 2000, 2001). Recorded from Maryland by Richardson (1905), Hornung and Szlávecz (2003), Norden (2008) and Maryland Biodiversity Project. Habitat: Diverse mesic terrestrial habitats.

*Porcelliospinicornis* Say, 1818 (Figure 4C)

With features of the family, also the following: Body length up to 12 mm. Body outline similar to Figure 4A. Head with frontal margin produced medially into broad convex to rectangular projection that extends laterally almost to the medial edge of large anterolateral lobes; each anterolateral lobe about a third the width of head; in dorsal view, the median and anterolateral lobes join at V-shaped notch. Head dark, pereon generally lighter than head but with dark median stripe; in life, dark stripe bordered laterally by bright yellow markings. Source: Hopkin (2014). U.S. Distribution: Introduced. Northeastern and northern Great Lake states, Nebraska, Tennessee, Arkansas (Jass and Klausmeier 2000, 2001); also North Dakota, Iowa (BugGuide), and Ohio (iNaturalist). Not yet recorded from Maryland. Habitat: Moist substrates and associated vertical surfaces.

*Porcellionidespruinosus* (Brandt, 1833) (Figure 4B)

With features of the family, also the following: Body length up to 12 mm. Body often with frosted gray-white appearance due to layer of microscopic beads, structural color that varies with lighting and timing of molt cycle (Hadley and Hendricks 1985). When coating absent, color varying but often red-brown, sometimes with light wavy markings. Head with frontal margin lacking median projection. Anterolateral lobes weakly developed. Antennae with articles IV and V with terminal white bands; first flagellar article about twice as long as second. Pleon abruptly narrower than pereon. Sources: Richardson (1905), Schultz (1982) and Hopkin (2014). U.S. Distribution: Introduced. Recorded throughout the contiguous 48 US states. Recorded in Maryland by Richardson (1905) (as *Metoponorthuspruinosus*); *Porcellionides* sp. recorded by Maryland Biodiversity Project. Habitat: Rotting logs, dung, etc.

### Family Scyphacidae

*Scyphacellaarenicola* Smith, 1873 (Figure 1E)

Body length up to 5 mm. Compound eyes present. Antennae with four flagellar articles that decrease in length distad, base of flagellum only slightly narrower than preceding article. Head without frontal margin, no clear border between cephalic dorsum and frontal lamina. Pereon with numerous scales; cuticle tuberculate, each tubercle tipped with small spine. Source: Richardson (1905), Schultz (1972). U.S. Distribution: Native. Massachusetts, Rhode Island, Connecticut, New Jersey, Delaware, and Florida (Jass and Klausmeier 2000, 2001). Reported from Maryland (Dorchester Co.) by Richardson (1905) and Schultz (1972) at Choptank River (not “Cleoptauk River”). Habitat: Littoral; marine sand beaches above high water mark.

### Family Trachelipodidae

*Trachelipusrathkii* (Brandt, 1833) (Figure 4D)

Body length up to 15 mm. Body outline similar to Figure 4A in dorsal perspective. Compound eyes present. Antennae with two distinct flagellar articles. Pereon with low bumps or tubercles; surface usually mottled dark brown, red brown and tan, with distinct lateral tan patches at base of epimera creating a pair of broken lateral light lines. Five pairs of lungs. Uropods projecting beyond pleotelson and general body outline. Sources: Richardson (1905) and Hopkin (2014). U.S. Distribution: Introduced. Maine south to North Carolina and west to Wisconsin and Arkansas; also Washington State (Jass and Klausmeier 2001). Recorded in Maryland by Norden (2008), Hornung et al. (2015), BugGuide, iNaturalist, and Maryland Biodiversity Project. Habitat: Moist soil, leaf litter, etc.

### Family Trichoniscidae (*Haplophthalmus*, *Hyloniscus*, *Miktoniscus*, *Trichoniscus*) (Figure 2)

Body length < 8 mm. Each eye with one ocellus (*Haplophthalmus*, *Hyloniscus*, *Miktoniscus*) or three ocelli (*Trichoniscus*). Antenna ending in a narrow, tapering, pointed flagellum (Figure 2A–C) comprising up to six articles when observed using high magnification. Lungs absent. Pleotelson with terminus truncate or with median notch or concavity (emarginate), not pointed (Figure 2A–C).

*Haplophthalmusdanicus* Budde-Lund, 1880 (Figure 2B)

With features of the family, also the following: Body length up to 4 mm. Each eye with one ocellus. Antennae with three flagellar articles observable with high magnification. Cuticle lacking dark pigments; translucent, white or cream. Head densely tuberculate dorsally, tubercles conical. Tergites of pereon armed with low longitudinal crests with roughened and/or tuberculate dorsal surfaces. Pleon segments III–V with prominent epimera; outline of pleon not abruptly narrower than pereon. Sources: Richardson (1905), Schultz (1982) and Hopkin (2014). U.S. Distribution: Introduced. Known from the eastern and southwestern states (Jass and Klausmeier 2000, 2001). Recorded in Maryland by Lohmander (1927), Hornung and Szlávecz (2003), Norden (2008) and Hornung et al. (2015). Habitat: Moist litter and debris.

*Hyloniscusriparius* (C. L. Koch, 1838)

With features of the family, also the following: Body length up to 7 mm. Body outline similar to Figure 2A. Each eye with one ocellus. Antennae with six flagellar articles observable with high magnification. Dorsum smooth, without tubercles or other sculpture. Pleon abruptly narrower than pereon. Male with hook on third segment (merus) of pereopod 7. U.S. Source: Richardson (1905) and Schultz (1965). U.S. Distribution: Introduced. Recorded from Maryland by Hornung and Szlávecz (2003), Norden (2008) and Hornung et al. (2015). Habitat: Moist to wet soil or litter, flood plains.

*Miktoniscusspinosus* (Say, 1818) (= *M.halophilus* Blake, 1931) (Figure 2C–E)

With features of the family, also the following: Body length up to 5 mm. Each eye with one ocellus. Antennae with four flagellar articles observable with high magnification. Dorsum of pereon with transverse to roughly transverse rows of tubercles. Pleon abruptly narrower than pereon. In male: Pleopod I with exopodite an elongate plate narrowing distally to a terminal point, exopodite only slightly shorter than endopodite; endopodite cylindrical, terminating with a distinct medial bend (Figure 2D). Pleopod II with plate-like endopodite short, with small distomedial lobe; terminus of endopodite spatulate with square apical border (Figure 2E). Source: Schultz (1976). U.S. Distribution: Native. Coastal reed marshes from Massachusetts south to Georgia (Schultz 1976, 2001); unidentified *Miktoniscus* in DC (BugGuide). Recorded from “edge of an estuary” in Calvert County, Maryland by Schultz (1976) as *M.halophilus*. Habitat: Brackish and estuarine marshes along the Atlantic Coast, also moist leaf litter of inland forests in river bottoms and near streams in piedmont of North Carolina (Schultz 1976).

*Miktoniscusmedcofi* (Van Name, 1940) (Figure 2C, F, G)

With features of the family, also the following: Body length up to 5 mm. Each eye with one ocellus. Antennae with four flagellar articles visible with high magnification. Dorsum of pereon with transverse to roughly transverse rows of tubercles. Pleon abruptly narrower than pereon. In male: Pleopod I with plate-like exopodite tapering distally to broadly rounded apex; endopodite tapering and flattened distally but resulting lamella with central longitudinal “mid-rib”, terminus with file-like striations under high magnification (Figure 2F). Pleopod II with plate-like exopodite comparatively long with long distomedial lobe; terminus of endopodite not spatulate (Figure 2G). Sources: Van Name (1940) and Schultz (1976). U.S. Distribution: Native. New York south to Florida west to central states (Jass and Klausmeier 2000, 2001), including Texas (Hutchins and Drukker 2016); unidentified *Miktoniscus* in DC (BugGuide). Not yet recorded from Maryland, but its presence in adjacent areas suggests that it occurs in the state. Habitat: Moist soil, under logs, caves.

*Trichoniscuspusillus* Brandt, 1833 (Figure 2A)

With features of the family, also the following: Body length up to 5 mm. Each eye with three ocelli. Antenna with four or five flagellar articles visible using high magnification. Dorsal cuticle smooth, with reddish to purplish pigments. Pleon abruptly narrower than pereon. Posterior margin of pleotelson with median concavity (emarginate). Sources: Richardson (1905), Schultz (1982) and Hopkin (2014). U.S. Distribution: Introduced. Northeastern states from Maine south to North Carolina and west to Wisconsin and Arkansas; also Washington State (Jass and Klausmeier 2001). Recorded in Maryland by Hornung and Szlávecz (2003) and Norden (2008). Habitat: Moist soil and litter.
